# Supplementary material for: An artificial intelligence‐assisted diagnostic platform for rapid near‐patient hematology
Source: Am J Hematol. 2021 Aug 2;96(10):1264–74. doi: 10.1002/ajh.26295 (PMC9290600; doi:10.1002/ajh.26295)
Supplement: Supplementary file 1 — Appendix S1. Supporting Information [file AJH-96-1264-s001.docx]

# Supplementary Materials for:

An Artificial Intelligence-Assisted Diagnostic Platform for Rapid Near-Patient Hematology

5 Supplemental Tables

4 Supplemental Figures

**Supplemental Table S1:** Summary of the method comparison study comparing the Sight OLO and the Sysmex XN and presented in Figure 2.

| **Measurand** | **N** | **Results Range** | **Correlation Coefficient (r)** | **Slope (95% CI)** | **Intercept (95% CI)** | **Median Bias** | **Median Relative Bias (%)** |
| --- | --- | --- | --- | --- | --- | --- | --- |
| WBC x10^3^/µL | 608 | 0.30 to 97.16 | 0.997 | 1.011  (1.003, 1.020) | 0.033  (-0.016, 0.082) | 0.1 | 1.6 |
| RBC x10^6^/µL | 657 | 1.86 to 7.69 | 0.991 | 1.019  (1.008, 1.030) | 0.089  (0.043, 0.129) | 0.16 | 4.1 |
| PLT x10^3^/µL | 624 | 22 to 985 | 0.985 | 1.006  (0.994, 1.021) | 8.937  (6.103, 11.801) | 10 | 5.2 |
| HGB g/dL | 673 | 4.9 to 21.2 | 0.990 | 1.031  (1.020, 1.042) | -0.028  (-0.150, 0.104) | 0.3 | 2.9 |
| HCT | 657 | 15.2 to 63.7 | 0.982 | 1.030  (1.014, 1.045) | -0.639  (-1.214, -0.044) | 0.4 | 1.3 |
| MCV fL | 657 | 57.3 to 121.2 | 0.941 | 0.889  (0.862, 0.916) | 7.489  (5.119, 9.821) | -2.3 | -2.6 |
| RDW | 647 | 10.6 to 29.4 | 0.939 | 1.000  (0.980, 1.036) | -0.100  (-0.607, 0.171) | -0.1 | -0.7 |
| MCH pg | 652 | 14.9 to 42.0 | 0.978 | 1.000  (0.986, 1.007) | -0.400  (-0.583, 0.046) | -0.4 | -1.3 |
| MCHC g/dL | 652 | 26.0 to 36.6 | 0.693 | 0.667  (0.625, 0.731) | 11.567  (9.431, 12.944) | 0.5 | 1.5 |
| NEUT% | 419 | 0.4 to 96.4 | 0.989 | 0.994  (0.980, 1.008) | 0.980  (0.051, 1.861) | 0.5 | 0.9 |
| NEUT# x10^3^/µL | 412 | 0.01 to 52.59 | 0.995 | 1.023  (1.011, 1.033) | 0.020  (-0.014, 0.070) | 0.12 | 2.9 |
| LYMPH% | 423 | 0.9 to 99.6 | 0.992 | 1.000  (0.989, 1.013) | 0.800  (0.452, 1.033) | 0.8 | 3.3 |
| LYMPH# x10^3^/µL | 415 | 0.02 to 8.44 | 0.987 | 1.025  (1.007, 1.044) | 0.050  (0.022, 0.083) | 0.09 | 5.7 |
| MONO% | 423 | 0.0 to 23.4 | 0.886 | 0.962  (0.910, 1.000) | -0.646  (-0.900, -0.264) | -0.9 | -12.5 |
| MONO# x10^3^/µL | 415 | 0.00 to 3.74 | 0.964 | 0.982  (0.941, 1.000) | -0.049  (-0.060, -0.027) | -0.06 | -10.9 |
| EOS% | 436 | 0.0 to 33.3 | 0.979 | 1.023  (1.000, 1.071) | 0.170  (0.100, 0.200) | 0.2 | 11.1 |
| EOS# x10^3^/µL | 427 | 0.00 to 4.24 | 0.986 | 1.042  (1.000, 1.094) | 0.014  (0.009, 0.020) | 0.02 | 15.0 |
| BASO% | 438 | 0.0 to 3.1 | 0.670 | 1.500  (1.333, 1.667) | -0.250  (-0.300, -0.167) | 0 | 0.0 |
| BASO# x10^3^/µL | 429 | 0.00 to 0.34 | 0.641 | 1.333  (1.200, 1.500) | -0.010  (-0.015, -0.006) | 0 | 0.0 |

BASO, basophil; CI, confidence interval; EOS, eosinophil; HCT, hematocrit; HGB, hemoglobin; LYMPH, lymphocyte; MCHC, mean corpuscular hemoglobin concentration; MCH, mean corpuscular hemoglobin; MCV, mean corpuscular volume; MONO, monocyte; NEUT, neutrophil; PLT, platelet; WBC, white blood cell; RBC, red blood cell; RDW, red blood cell distribution width.

**Supplemental Table S2:** Summary of the method comparison study including results invalidated by OLO (corresponding to Supplemental Figure 1).

| **Measurand** | **N** | **Results Range** | **Correlation Coefficient (r)** | **Slope (95% CI)** | **Intercept (95% CI)** | **Median Bias** | **Median Relative Bias (%)** |
| --- | --- | --- | --- | --- | --- | --- | --- |
| WBC x10^3^/µL | 670 | 0.3 to 97.16 | 0.997 | 1.009  (1.001, 1.018) | 0.034  (-0.012, 0.083) | 0.09 | 1.5 |
| RBC x10^6^/µL | 672 | 1.86 to 7.69 | 0.99 | 1.017  (1.006, 1.027) | 0.098  (0.053, 0.139) | 0.16 | 4.0 |
| PLT x10^3^/µL | 637 | 22.0 to 1017.0 | 0.985 | 1.004  (0.992, 1.018) | 9.413  (6.67, 12.248) | 10 | 5.2 |
| HGB g/dL | 673 | 4.9 to 21.2 | 0.99 | 1.031  (1.02, 1.042) | -0.028  (-0.15, 0.104) | 0.3 | 2.9 |
| HCT | 672 | 15.2 to 63.7 | 0.982 | 1.028  (1.012, 1.044) | -0.567  (-1.152, 0.015) | 0.4 | 1.3 |
| MCV fL | 671 | 57.3 to 121.2 | 0.943 | 0.889  (0.863, 0.915) | 7.444  (5.142, 9.725) | -2.3 | -2.6 |
| RDW | 671 | 10.6 to 29.4 | 0.943 | 1.0  (0.979, 1.031) | -0.1  (-0.537, 0.196) | -0.1 | -0.7 |
| MCH pg | 667 | 14.9 to 42.0 | 0.978 | 1.0  (0.987, 1.007) | -0.4  (-0.592, 0.004) | -0.4 | -1.3 |
| MCHC g/dL | 667 | 26.0 to 37.2 | 0.7 | 0.683  (0.633, 0.738) | 10.998  (9.193, 12.66) | 0.5 | 1.5 |
| NEUT% | 551 | 0.4 to 96.4 | 0.987 | 0.995  (0.982, 1.008) | 1.029  (0.136, 1.841) | 0.7 | 1.0 |
| NEUT# x10^3^/µL | 551 | 0.01 to 52.59 | 0.995 | 1.025  (1.016, 1.034) | 0.016  (-0.01, 0.052) | 0.12 | 3.0 |
| LYMPH% | 576 | 0.9 to 99.6 | 0.99 | 1.0  (0.988, 1.009) | 0.8  (0.588, 1.083) | 0.8 | 3.8 |
| LYMPH# x10^3^/µL | 576 | 0.02 to 8.44 | 0.984 | 1.022  (1.006, 1.038) | 0.056  (0.034, 0.084) | 0.09 | 5.9 |
| MONO% | 576 | 0.0 to 37.1 | 0.925 | 0.917  (0.879, 0.957) | -0.221  (-0.526, 0.088) | -0.9 | -10.9 |
| MONO# x10^3^/µL | 576 | 0.0 to 7.86 | 0.951 | 1.0 (0.958, 1.008) | -0.06  (-0.062, -0.032) | -0.06 | -10.3 |
| EOS% | 606 | 0.0 to 56.4 | 0.983 | 1.0  (0.976, 1.0) | 0.2  (0.2, 0.213) | 0.2 | 13.5 |
| EOS# x10^3^/µL | 606 | 0.0 to 34.55 | 0.999 | 1.0  (1.0, 1.045) | 0.02  (0.01, 0.02) | 0.02 | 16.0 |
| BASO% | 644 | 0.0 to 5.1 | 0.629 | 1.333  (1.2, 1.5) | -0.167  (-0.25, -0.14) | 0 | -8.1 |
| BASO# x10^3^/µL | 644 | 0.0 to 1.36 | 0.713 | 1.25  (1.043, 1.333) | -0.01  (-0.013, -0.001) | 0 | -0.1 |

BASO, basophil; CI, confidence interval; EOS, eosinophil; HCT, haematocrit; HGB, haemoglobin; LYMPH, lymphocyte; MCHC, mean corpuscular haemoglobin concentration; MCH, mean corpuscular haemoglobin; MCV, mean corpuscular volume; MONO, monocyte; NEUT, neutrophil; PLT, platelet; WBC, white blood cell; RBC, red blood cell; RDW, red blood cell distribution width.

**Supplemental Table S3:** Sensitivity and specificity for WBC morphological abnormalities (blasts, immature granulocytes, nucleated RBCs, and atypical lymphocytes) compared to light microscopy.

|  | Flagged by OLO | Not flagged by OLO |  | % | 95% CI | |
| --- | --- | --- | --- | --- | --- | --- |
| Positive by  light microscopy | 93 | 7 | Sensitivity (PPA) | 93.0% | 86.1% | 97.1% |
| Negative by  light microscopy | 21 | 87 | Specificity (NPA) | 80.6% | 71.8% | 87.5% |
|  |  |  | Overall Agreement | 86.5% | 81.1% | 90.9% |

## Supplemental Table S4: Results of the reproducibility study, in which three levels of commercial control material (low, normal and high) were analyzed for all reported parameters. 240 samples were included for each level of control in these studies.

| **Measurand** | **Level** | **Mean** | **Within run** | | **Between Run** | | **Between Day** | | **Between instrument** | | **Between Site** | | **Total** | |
| --- | --- | --- | --- | --- | --- | --- | --- | --- | --- | --- | --- | --- | --- | --- |
|  |  |  | **SD** | **CV%** | **SD** | **CV%** | **SD** | **CV%** | **SD** | **CV%** | **SD** | **CV%** | **SD** | **CV%** |
| WBC | Low | 3.33 | 0.17 | 5.2 | 0.06 | 1.7 | 0.00 | 0.0 | 0.11 | 3.3 | 0.00 | 0.0 | 0.21 | 6.4 |
|  | Normal | 8.19 | 0.26 | 3.2 | 0.09 | 1.1 | 0.00 | 0.0 | 0.17 | 2.1 | 0.00 | 0.0 | 0.32 | 4.0 |
|  | High | 23.02 | 0.62 | 2.7 | 0.25 | 1.1 | 0.10 | 0.4 | 0.39 | 1.7 | 0.00 | 0.0 | 0.78 | 3.4 |
| RBC | Low | 2.61 | 0.04 | 1.5 | 0.04 | 1.7 | 0.00 | 0.0 | 0.07 | 2.8 | 0.00 | 0.0 | 0.09 | 3.6 |
|  | Normal | 5.14 | 0.05 | 0.9 | 0.04 | 0.8 | 0.00 | 0.0 | 0.10 | 1.9 | 0.00 | 0.0 | 0.12 | 2.2 |
|  | High | 5.91 | 0.06 | 1.0 | 0.03 | 0.6 | 0.02 | 0.4 | 0.10 | 1.7 | 0.00 | 0.0 | 0.12 | 2.1 |
| PLT | Low | 70.7 | 5.1 | 7.2 | 1.0 | 1.4 | 0.0 | 0.0 | 1.5 | 2.1 | 3.1 | 4.4 | 6.2 | 8.8 |
|  | Normal | 218.2 | 9.4 | 4.3 | 2.5 | 1.2 | 2.1 | 0.9 | 3.1 | 1.4 | 2.7 | 1.2 | 10.8 | 4.9 |
|  | High | 432.6 | 19.7 | 4.5 | 7.0 | 1.6 | 6.9 | 1.6 | 10.9 | 2.5 | 0.0 | 0.0 | 24.5 | 5.7 |
| HGB | Low | 7.05 | 0.10 | 1.4 | 0.09 | 1.2 | 0.00 | 0.0 | 0.10 | 1.4 | 0.00 | 0.0 | 0.16 | 2.3 |
|  | Normal | 14.41 | 0.10 | 0.7 | 0.05 | 0.4 | 0.02 | 0.1 | 0.11 | 0.7 | 0.11 | 0.8 | 0.19 | 1.3 |
|  | High | 17.32 | 0.13 | 0.7 | 0.06 | 0.3 | 0.05 | 0.3 | 0.11 | 0.6 | 0.20 | 1.2 | 0.27 | 1.6 |
| HCT | Low | 21.15 | 0.39 | 1.9 | 0.25 | 1.2 | 0.10 | 0.5 | 0.51 | 2.4 | 0.00 | 0.0 | 0.70 | 3.3 |
|  | Normal | 43.99 | 0.60 | 1.4 | 0.35 | 0.8 | 0.00 | 0.0 | 0.81 | 1.8 | 0.00 | 0.0 | 1.06 | 2.4 |
|  | High | 56.55 | 0.84 | 1.5 | 0.44 | 0.8 | 0.17 | 0.3 | 0.82 | 1.5 | 0.00 | 0.0 | 1.26 | 2.2 |
| MCV | Low | 80.91 | 0.84 | 1.0 | 0.74 | 0.9 | 0.00 | 0.0 | 0.79 | 1.0 | 0.00 | 0.0 | 1.37 | 1.7 |
|  | Normal | 85.62 | 0.81 | 1.0 | 0.72 | 0.8 | 0.00 | 0.0 | 0.98 | 1.1 | 0.00 | 0.0 | 1.46 | 1.7 |
|  | High | 95.66 | 0.86 | 0.9 | 0.52 | 0.5 | 0.00 | 0.0 | 1.51 | 1.6 | 0.00 | 0.0 | 1.82 | 1.9 |
| RDW | Low | 14.20 | 0.26 | 1.8 | 0.07 | 0.5 | 0.01 | 0.1 | 0.03 | 0.2 | 0.16 | 1.2 | 0.32 | 2.2 |
|  | Normal | 13.17 | 0.19 | 1.5 | 0.00 | 0.0 | 0.01 | 0.1 | 0.06 | 0.5 | 0.07 | 0.6 | 0.22 | 1.6 |
|  | High | 12.57 | 0.19 | 1.5 | 0.00 | 0.0 | 0.00 | 0.0 | 0.07 | 0.6 | 0.07 | 0.5 | 0.21 | 1.7 |
| MCH | Low | 26.99 | 0.19 | 0.7 | 0.17 | 0.6 | 0.05 | 0.2 | 0.49 | 1.8 | 0.00 | 0.0 | 0.55 | 2.1 |
|  | Normal | 28.04 | 0.18 | 0.6 | 0.15 | 0.5 | 0.00 | 0.0 | 0.44 | 1.6 | 0.00 | 0.0 | 0.50 | 1.8 |
|  | High | 29.30 | 0.22 | 0.7 | 0.04 | 0.1 | 0.05 | 0.2 | 0.46 | 1.6 | 0.00 | 0.0 | 0.52 | 1.8 |
| MCHC | Low | 33.37 | 0.38 | 1.1 | 0.26 | 0.8 | 0.00 | 0.0 | 0.60 | 1.8 | 0.00 | 0.0 | 0.76 | 2.3 |
|  | Normal | 32.76 | 0.39 | 1.2 | 0.23 | 0.7 | 0.02 | 0.1 | 0.46 | 1.4 | 0.00 | 0.0 | 0.65 | 2.0 |
|  | High | 30.63 | 0.40 | 1.3 | 0.15 | 0.5 | 0.00 | 0.0 | 0.32 | 1.0 | 0.31 | 1.0 | 0.62 | 2.0 |
| NEUT% | Low | 50.32 | 2.44 | 4.9 | 0.00 | 0.0 | 0.46 | 0.9 | 0.43 | 0.9 | 0.97 | 1.9 | 2.70 | 5.4 |
|  | Normal | 41.73 | 1.62 | 3.9 | 0.00 | 0.0 | 0.00 | 0.0 | 0.21 | 0.5 | 0.00 | 0.0 | 1.64 | 3.9 |
|  | High | 54.35 | 1.05 | 1.9 | 0.16 | 0.3 | 0.19 | 0.4 | 0.48 | 0.9 | 0.63 | 1.2 | 1.34 | 2.5 |
| NEUT# | Low | 1.68 | 0.12 | 7.0 | 0.02 | 1.4 | 0.03 | 1.7 | 0.05 | 3.2 | 0.00 | 0.0 | 0.13 | 8.0 |
|  | Normal | 3.42 | 0.16 | 4.7 | 0.05 | 1.3 | 0.00 | 0.0 | 0.06 | 1.9 | 0.00 | 0.0 | 0.18 | 5.2 |
|  | High | 12.51 | 0.39 | 3.1 | 0.14 | 1.2 | 0.10 | 0.8 | 0.26 | 2.1 | 0.16 | 1.3 | 0.53 | 4.2 |
| LYMPH% | Low | 17.62 | 1.69 | 9.6 | 0.29 | 1.6 | 0.00 | 0.0 | 0.00 | 0.0 | 0.00 | 0.0 | 1.71 | 9.7 |
|  | Normal | 36.41 | 1.42 | 3.9 | 0.00 | 0.0 | 0.30 | 0.8 | 0.00 | 0.0 | 0.29 | 0.8 | 1.48 | 4.1 |
|  | High | 25.30 | 0.91 | 3.6 | 0.00 | 0.0 | 0.17 | 0.7 | 0.07 | 0.3 | 0.00 | 0.0 | 0.93 | 3.7 |
| LYMPH# | Low | 0.59 | 0.06 | 10.4 | 0.02 | 3.7 | 0.00 | 0.0 | 0.01 | 2.5 | 0.00 | 0.0 | 0.07 | 11.4 |
|  | Normal | 2.98 | 0.15 | 5.2 | 0.01 | 0.5 | 0.02 | 0.7 | 0.08 | 2.5 | 0.00 | 0.0 | 0.17 | 5.8 |
|  | High | 5.82 | 0.28 | 4.7 | 0.00 | 0.0 | 0.03 | 0.6 | 0.09 | 1.5 | 0.00 | 0.0 | 0.29 | 5.0 |
| MONO% | Low | 12.21 | 1.53 | 12.5 | 0.00 | 0.0 | 0.22 | 1.8 | 0.73 | 6.0 | 0.59 | 4.9 | 1.81 | 14.8 |
|  | Normal | 8.44 | 0.89 | 10.6 | 0.14 | 1.6 | 0.07 | 0.9 | 0.30 | 3.6 | 0.43 | 5.1 | 1.05 | 12.4 |
|  | High | 11.88 | 0.76 | 6.4 | 0.00 | 0.0 | 0.00 | 0.0 | 0.42 | 3.6 | 0.72 | 6.0 | 1.12 | 9.5 |
| MONO# | Low | 0.41 | 0.05 | 13.4 | 0.00 | 0.0 | 0.00 | 0.0 | 0.04 | 9.3 | 0.01 | 1.5 | 0.07 | 16.4 |
|  | Normal | 0.69 | 0.08 | 11.2 | 0.02 | 2.3 | 0.00 | 0.0 | 0.04 | 5.2 | 0.03 | 3.7 | 0.09 | 13.1 |
|  | High | 2.73 | 0.19 | 7.0 | 0.03 | 0.9 | 0.00 | 0.0 | 0.12 | 4.4 | 0.14 | 5.0 | 0.26 | 9.7 |
| EOS% | Low | 11.75 | 1.69 | 14.4 | 0.30 | 2.6 | 0.00 | 0.0 | 0.00 | 0.0 | 0.41 | 3.5 | 1.76 | 15.0 |
|  | Normal | 7.09 | 0.85 | 11.9 | 0.19 | 2.7 | 0.00 | 0.0 | 0.08 | 1.1 | 0.14 | 2.0 | 0.88 | 12.5 |
|  | High | 3.94 | 0.47 | 11.9 | 0.00 | 0.0 | 0.00 | 0.0 | 0.00 | 0.0 | 0.00 | 0.0 | 0.47 | 11.9 |
| EOS# | Low | 0.39 | 0.06 | 16.2 | 0.01 | 2.2 | 0.00 | 0.0 | 0.02 | 5.3 | 0.01 | 1.3 | 0.07 | 17.2 |
|  | Normal | 0.58 | 0.07 | 12.4 | 0.02 | 3.9 | 0.00 | 0.0 | 0.02 | 2.8 | 0.00 | 0.0 | 0.08 | 13.3 |
|  | High | 0.91 | 0.11 | 12.6 | 0.00 | 0.0 | 0.01 | 1.4 | 0.01 | 0.6 | 0.00 | 0.0 | 0.12 | 12.7 |
| BASO% | Low | 8.12 | 1.23 | 15.1 | 0.00 | 0.0 | 0.20 | 2.5 | 0.03 | 0.3 | 0.21 | 2.6 | 1.26 | 15.5 |
|  | Normal | 6.33 | 0.75 | 11.9 | 0.00 | 0.0 | 0.00 | 0.0 | 0.00 | 0.0 | 0.16 | 2.5 | 0.77 | 12.1 |
|  | High | 4.54 | 0.48 | 10.5 | 0.13 | 2.8 | 0.00 | 0.0 | 0.00 | 0.0 | 0.12 | 2.5 | 0.51 | 11.2 |
| BASO# | Low | 0.27 | 0.04 | 15.4 | 0.01 | 2.3 | 0.01 | 1.9 | 0.00 | 1.8 | 0.00 | 1.0 | 0.04 | 15.8 |
|  | Normal | 0.52 | 0.07 | 12.6 | 0.00 | 0.0 | 0.00 | 0.0 | 0.01 | 1.7 | 0.01 | 2.6 | 0.07 | 12.9 |
|  | High | 1.04 | 0.11 | 10.7 | 0.04 | 3.5 | 0.00 | 0.0 | 0.03 | 3.1 | 0.02 | 2.4 | 0.12 | 11.9 |

BASO, basophil; CI, confidence interval; EOS, eosinophil; HCT, hematocrit; HGB, hemoglobin; LYMPH, lymphocyte; MCHC, mean corpuscular hemoglobin concentration; MONO, Monocyte; MCH, mean corpuscular hemoglobin; MCV, mean corpuscular volume; NEUT, neutrophil; PLT, platelet; RBC, red blood cell; RDW, red blood cell distribution width; WBC, white blood cell.

**Supplemental Table S5: Finger-Prick vs. Capillary Matrix Study Results**

| **Measurand** | **N** | **Finger** | | | **Capillary** | | | **Slope** | **Slope CI** | **Intercept** | **Intercept CI** | **r** | **Bias** |
| --- | --- | --- | --- | --- | --- | --- | --- | --- | --- | --- | --- | --- | --- |
|  |  | **Mean** | **Min** | **Max** | **Mean** | **Min** | **Max** |  |  |  |  |  |  |
| **WBC**  **(x10^3^/μL)** | 40 | 8.08 | 3.75 | 12.16 | 8.01 | 3.8 | 12.51 | 0.97 | (0.89, 1.06) | 0.3 | (-0.3, 0.9) | 0.97 | 1.4% |
| **RBC**  **(x10^6^/μL)** | 40 | 4.797 | 3.61 | 6.14 | 5.081 | 4.07 | 6.23 | 0.99 | (0.87, 1.10) | -0.2 | (-0.8, 0.4) | 0.96 | -5.3% |
| **PLT**  **(x10^3^/μL)** | 40 | 262.9 | 138 | 431 | 264.2 | 148 | 412 | 1.02 | (0.87, 1.17) | -7 | (-44, 36) | 0.92 | -0.7% |
| **HGB**  **(g/dL)** | 40 | 13.56 | 10.5 | 17.5 | 14.35 | 12.0 | 17.8 | 1.02 | (0.90, 1.16) | -1.0 | (-3.1, 0.7) | 0.95 | -5.2% |
| **MCV**  **(fL)** | 40 | 84.43 | 77.5 | 93.4 | 84.35 | 78.2 | 92.6 | 1.01 | (0.89, 1.10) | -0.8 | (-7.8, 9.6) | 0.95 | 0.3% |
| **RDW**  **(%)** | 40 | 12.58 | 11.2 | 13.7 | 12.48 | 11.2 | 14.1 | 0.94 | (0.73, 1.13) | 0.9 | (-1.5, 3.4) | 0.85 | 1.2% |
| **MCH**  **(pg)** | 40 | 28.31 | 25.2 | 31.6 | 28.28 | 25.2 | 31.4 | 1.00 | (0.95, 1.02) | 0.1 | (-0.4, 1.4) | 1.00 | 0.2% |
| **MCHC**  **(g/dL)** | 40 | 33.54 | 31.7 | 36.2 | 33.54 | 31.1 | 35.8 | 1.01 | (0.89, 1.17) | -0.5 | (-5.7, 3.8) | 0.92 | 0.0% |
| **NEUT#**  **(x10^3^/μL)** | 40 | 4.53 | 1.61 | 7.66 | 4.58 | 1.75 | 8.56 | 0.96 | (0.9, 1.06) | 0.1 | (-0.3, 0.4) | 0.97 | -1.4% |
| **LYMPH#**  **(x10^3^/μL)** | 40 | 2.74 | 1.58 | 5.21 | 2.58 | 1.4 | 3.94 | 1.04 | (0.93, 1.16) | 0.07 | (-0.23, 0.34) | 0.95 | 7.3% |
| **NEUT%** | 40 | 55.16 | 39.8 | 70.5 | 56.35 | 38.1 | 72 | 1.06 | (0.96, 1.17) | -4.5 | (-10.9, 1.5) | 0.95 | -1.1 |
| **LYMPH%** | 40 | 34.64 | 21.5 | 49.4 | 32.89 | 20.2 | 50.2 | 1.03 | (0.94, 1.13) | 0.7 | (-2.8, 3.4) | 0.96 | 1.6 |
| **MONO%** | 39 | 7.16 | 3.0 | 12.7 | 7.81 | 4.6 | 13.6 | 0.88 | (0.70, 1.11) | 0.2 | (-1.4, 1.6) | 0.80 | -0.6 |
| **MONO#**  **(x10^3^/μL)** | 39 | 0.57 | 0.22 | 1.13 | 0.62 | 0.31 | 0.93 | 0.93 | (0.73, 1.20) | 0.00 | (-0.16, 0.12) | 0.74 | -7.8% |
| **EOS%** | 40 | 2.56 | 0.1 | 13 | 2.54 | 0.2 | 12.1 | 1.01 | (0.92, 1.13) | 0.0 | (-0.2, 0.2) | 0.97 | 0.1 |
| **EOS#**  **(x10^3^/μL)** | 40 | 0.21 | 0.01 | 1.3 | 0.2 | 0.02 | 1.09 | 1.04 | (0.92, 1.13) | 0.00 | (-0.01, 0.02) | 0.98 | 0.01 |
| **BASO%** | 40 | 0.39 | 0 | 1.3 | 0.46 | 0 | 1.4 | 0.75 | (0.57, 1.0) | 0.0 | (-0.0, 0.1) | 0.74 | 0.0 |
| **BASO#**  **(x10^3^/μL)** | 40 | 0.03 | 0 | 0.09 | 0.04 | 0 | 0.11 | 0.83 | (0.67, 1.0) | 0.00 | (-0.01, 0.00) | 0.68 | -0.01 |

**
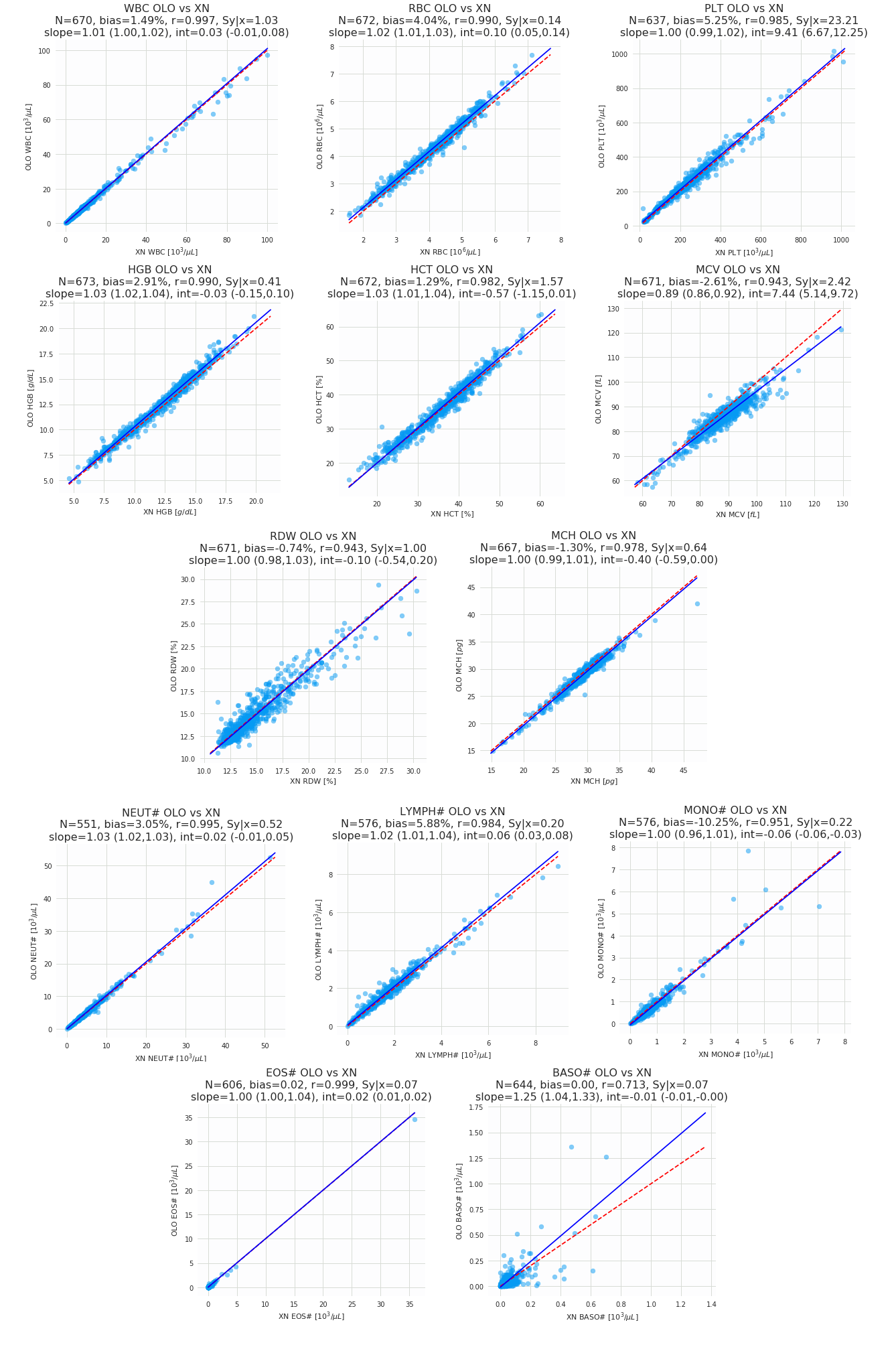
**

**Supplemental Figure S1.** Results of the method comparison study between the Sight OLO and the Sysmex XN hematology analyzers with measurands invalidated by the OLO included in the analysis (while measurands invalidated by the Sysmex still excluded). Graphs indicate Pearson correlation, slope and bias for each parameter. These results are tabulated in Supplemental Table 3.


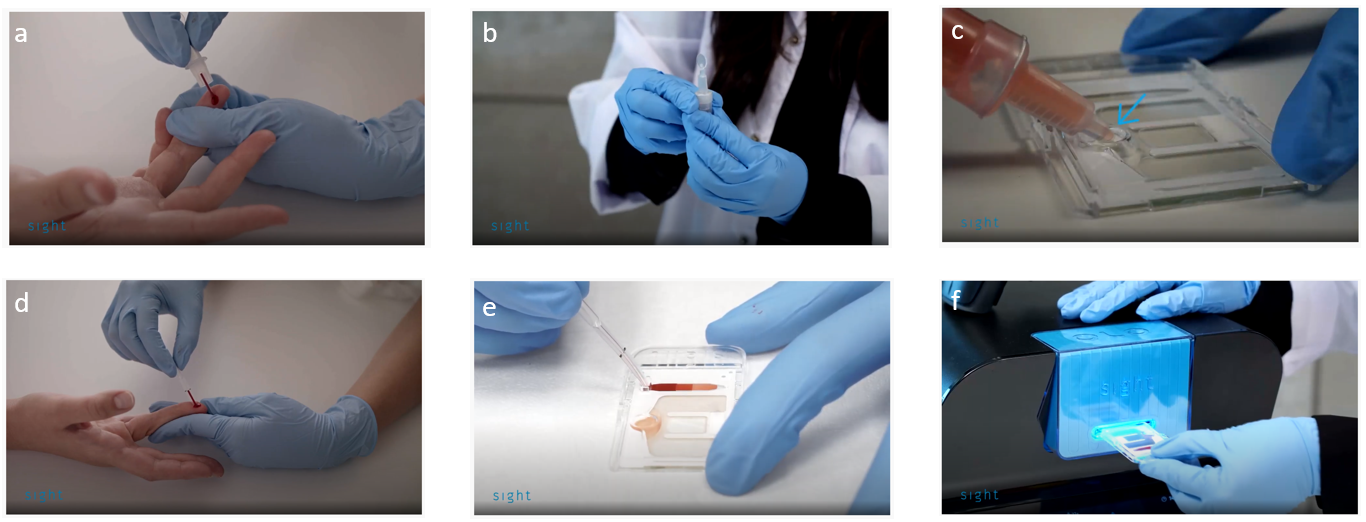


**Supplemental Figure S2.** Images depicting the Sight OLO sample preparation workflow for finger-prick samples: (a) collect sample using dropper cap; (b) insert dropper cap into mixing bottle; (c) following 10 inversions of the mixing bottle twist off cap and dispense into the imaging chamber; (d) collect sample using microcapillary; (e) dispense into hemoglobin measurement chamber; (f) insert cartridge into the device.


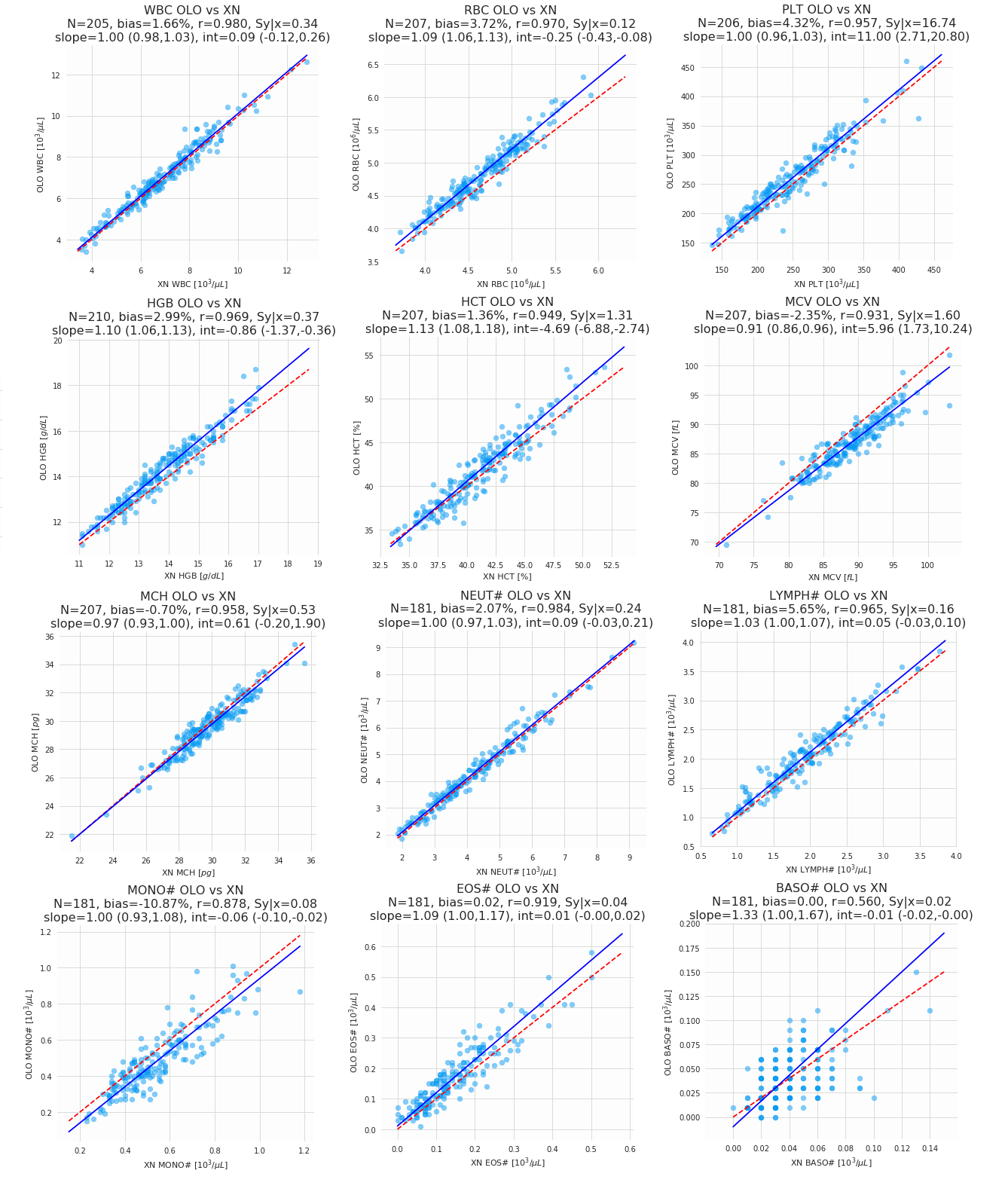


**Supplemental Figure S3.** Results of the method comparison study between the Sight OLO and the Sysmex XN hematology analyzers for apparently normal patients, i.e. (1) Lab results within lab reference range for the major measurands (WBC, RBC, HGB, PLT, #NEUT, #LYMPH) (2) no flags on lab automated hematology analyzer. Graphs indicate Pearson correlation, slope and bias for each parameter.


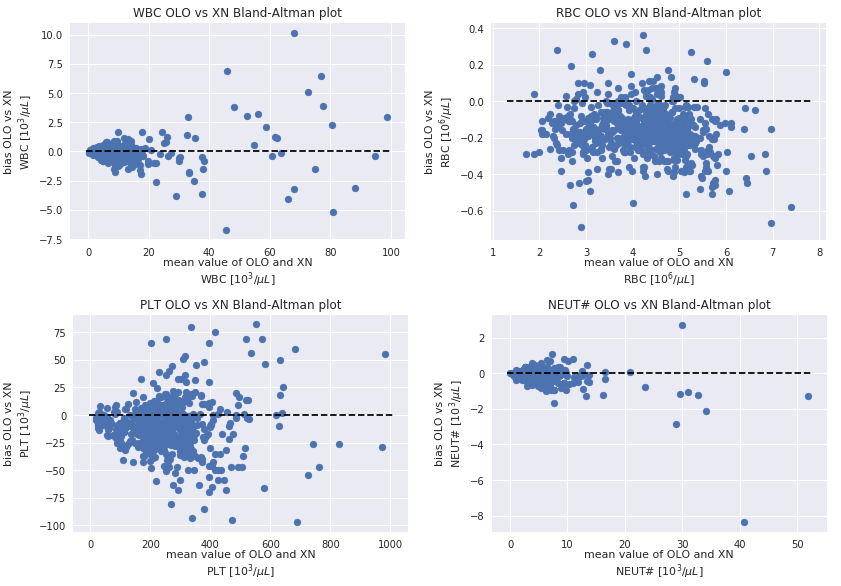


**Supplemental Figure S4.** Bland Altman of the results of method comparison study between the Sight OLO and the Sysmex XN hematology analyzers corresponding to Figure 3 for the main measurands.
